# Two-dimensional semantic morphological feature extraction and atlas construction of maize ear leaves

**DOI:** 10.3389/fpls.2025.1520297

**Published:** 2025-02-12

**Authors:** Hongli Song, Weiliang Wen, Ying Zhang, Yanxin Zhao, Xinyu Guo, Chunjiang Zhao

**Affiliations:** ^1^ College of Information Engineering, Northwest A&F University, Yangling, China; ^2^ Information Technology Research Center, Beijing Academy of Agriculture and Forestry Sciences, Beijing, China; ^3^ Beijing Key Lab of Digital Plant, National Engineering Research Center for Information Technology in Agriculture, Beijing, China; ^4^ Beijing Key Laboratory of Maize DNA (DeoxyriboNucleic Acid) Fingerprinting and Molecular Breeding, Maize Research Center, Beijing Academy of Agriculture and Forestry Sciences, Beijing, China

**Keywords:** maize, two-dimensional, leaf shape, phenotyping, semantic features

## Abstract

Maize ear leaves have important roles in photosynthesis, nutrient partitioning and hormone regulation. The morphological and structural variations observed in maize ear leaves are numerous and contribute significantly to the yield. Nevertheless, research on the fine-scale morphology of maize leaves is less, particularly the quantitative methods to characterize the morphology of leaves in two-dimensional (2D) space is absent. This makes it challenging to accurately identify 2D leaf shape of their cultivars. Therefore, this study presents the methods of 2D semantic morphological feature extraction and atlas construction, with the ear leaf in silking stage of maize association analysis population serving as an example. A three-dimensional (3D) digitizer was employed to obtain data from 1,431 leaves belonging to 518 inbred lines. The data was then processed using mesh subdivision and planar parameterization to create 2D leaf models with area-preserving characteristics. Additionally, averaged 2D leaf models of all the inbred lines were constructed, and 29 2D leaf features were quantified. Based on this, 11 features were extracted as semantic features of 2D leaf shape through clustering and correlation analysis. A comprehensive 2D leaf shape indicator *L*_2_ *_D_* based on the 11 semantic features was proposed, and a 2D leaf shape atlas was constructed in accordance with the *L*_2_ *_D_* ordering. Inbred line identification of 2D leaf shape in maize was achieved using the atlas. The results of maize leaf inbred line identification can determine the probability that the corresponding true inbred line ranked within the top 10 of the predicted results is 0.706, within the top 20 is 0.810, and within the top 45 is 0.900. This enables the generation of the corresponding maize 2D leaf shape through the matching of semantic features. The methodology presented in this study offers novel insights into the construction of semantic models for the morphology of maize and the identification of cultivars. It also provides a theoretical and technical foundation for the generation and drawing the leaf shape based on semantic 2D morphological and structural features.

## 1 Introduction

Plant architecture (Godin, 2000) is used to describe the observable characteristics of an individual or parts of plant. These characteristics encompass leaf shape, stem morphology, tassel structure, and root architecture, etc. Plant architecture exhibit variation among different species, different cultivars of the same species, and even among different individuals of the same cultivar. These differences are closely associated with the diverse genetic makeup. To a certain extent, plant architecture can be used as a group of indicators of the growth status of plants, which is of great significance to agricultural research and production. The plant architecture (Jafari et al., 2024) can be described by phenotypic big data, including topology, spatial geometric relationships of multiple organs in the multi-dimensional perspective. Leaf shape constitutes an essential element of plant architecture, encompassing aspects such as leaf spatial disposition, local blade folds, leaf margin amplitude, and others. The discrepancies in leaf shape directly influence the canopy structure and radiation utilization efficiency.

Maize is an important food and energy crop. The rapid acquisition of maize leaf shapes and analyzing the differences among cultivars is an important component of maize breeding (Tian et al., 2019) and dense density planting for high-yield cultivation (Li et al., 2021). In earlier research, simple one-dimensional (1D) measurement was the predominant way for obtaining leaf shape data, including leaf length, width, and aspect ratio. In further research of leaf shape, the researchers placed the maize leaf on a horizontal plane and attempted to flatten it as much as possible, then they used equipment, including RGB cameras (Han et al., 2014; Robil et al., 2021), hyperspectral cameras (Gao et al., 2021), etc., to capture images of the leaf from a position perpendicular to the horizontal plane. These images were used to extract 2D phenotypic features including the leaf area, leaf profile perimeter, etc. However, the process of flattening results loss 3D structural information, such as leaf bending and folding, which is not conducive to more in-depth research of the leaf shape from the 3D perspective. The advent of LiDAR, 3D digitizers, multi-view stereo (MVS), and other 3D data acquisition technologies has made the complete acquisition of the 3D leaf morphology feasible. The research of the leaf shape based on 3D data, in addition to obtaining the 1D and 2D phenotypic features, can also obtain 3D phenotypic features, such as the leaf curvature and the inclination angle (Bailey and Mahaffee, 2017; Hosoi et al., 2011). Nevertheless, the point cloud data obtained through LiDAR and other 3D techniques (Chen et al., 2023) necessitate intricate processing to generate 3D models comprising semantic information and to extract more 3D leaf shape features (Su et al., 2018; Wen et al., 2024b). In contrast, 3D digitizers are capable of directly acquiring data containing semantic information through the manual operation of the instrument acquisition method (Wen et al., 2021). The utilization of digitizers for the acquisition of 3D structural data pertaining to plants has been a subject of extensive research and development over a long time. This methodology has been employed in the investigation of diverse plant organs, including roots (Danjon and Reubens, 2008; Wu et al., 2015), leaves (Zheng et al., 2022), and the entire plant (Zheng et al., 2008).

The quantitative analysis of crop leaf shape features is closely related to the means of data acquisition. Wang et al. (2022) first segmented the foreground and background by converting color images to greyscale using an adaptive thresholding algorithm. After segmentation, features such as color and compactness were extracted in addition to conventional phenotypic features such as length, width and surface area. Wu et al. (2022) extracted 3D structure-related leaf features such as leaf inclination, leaf curvature, etc. from a 3D model of a maize leaf acquired by a 3D digitizer. Wen et al. (2024b) achieved mesh generation from the 3D point cloud of maize leaves by a series meshing technologies. The ARAP algorithm was used to convert the 3D mesh into 2D planar mesh, and finally both the 2D and 3D semantic leaf mesh model was generated. Based on the 3D semantic leaf model, 3D leaf area, leaf surface flatness and other phenotypic features that cannot be obtained based on 2D images can be extracted. Wen et al. (2024a) extracted 3D leaf features such as leaf inclination angle, blade-included angle, blade self-twisting, blade planarity, margin amplitude from a maize leaf model acquired by a 3D digitizer. In addition to maize leaf phenotypes, there are also related 3D quantitative analysis studies on wheat leaf phenotypes (Zheng et al., 2022). The current rapid development of 2D and 3D acquisition techniques and related quantitative analysis techniques has made it possible to acquire crop leaf-shape data in multiple dimensions and with high quality, thus enabling the completion of the analysis. The extraction of phenotypic features from crop leaves allows researchers to mine related physiological and ecological knowledge. In the previous research, Wang et al. (2022) validated the correlation between the sixth leaf sheath color phenotypic traits and the corresponding candidate genes by integrating the leaf sheath phenotypic data of the maize association analysis population (Yang et al., 2011) through GWAS. Wu et al. (2022) proposed a novel classification method based on the spatial morphology of the midvein curves in maize leaves. The analysis was also performed using GWAS to obtain the midvein curve of the leaves. GWAS was also employed to analyze the association between leaf midvein curves and genes.

Yang et al. (2011) constructed a large association panel in maize, and assembled a comprehensive maize association analysis population comprising 527 globally diverse lines representing tropical, subtropical, and temperate germplasm. The population is a collection of major inbred lines from around the world, which are believed to represent the principal morphological features of the maize genes and phenotypes. The use of the collection as a research object ensures comprehensive coverage of the diverse leaf shapes observed in maize leaves, thus facilitating subsequent genotype-phenotype correlation studies. To date, numerous genotype-phenotype related studies have been conducted on the maize association analysis population, with all of them yielding positive results (Gao et al., 2024; Song et al., 2024; Wang et al., 2024a, b; Wu et al., 2024; Xia et al., 2024; Yang et al., 2024).

At present, quantitative characterization methods for 2D leaf shape features of maize leaves are absent, and the interactions between leaf shape features remain unclear. Furthermore, there is a pressing need for big data analysis and knowledge mining for maize inbred populations within a phenomics perspective. The underlying laws governing leaf shape features embedded in maize association analysis population materials remain elusive. Consequently, it is not yet feasible to achieve inbred line identification and visualization based on leaf shape features. In this study, we utilize the maize ear position leaves of the population proposed by Yang et al. (2011) as a case study to investigate the quantitative extraction of maize 2D leaf shape features, the construction of a leaf shape atlas, and the identification inbred line according to the leaf shapes. It is anticipated to facilitate the extraction of knowledge from the big data pertaining to leaf shapes.

## 2 Materials and methods

### 2.1 Experimental design and data acquisition

The experiment was conducted at the Nanfan breeding station of the Maize Research Center, Beijing Academy of Agriculture and Forestry Sciences (BAAFS), in Yazhou District, Sanya City, Hainan Province, China (longitude 109.1832, latitude 18.3623). The material selected for analysis in this research was the collection mentioned in section 1 (Yang et al., 2011). The specific planting times and planting settings can be found in Wu et al. (2022).

**Figure 1:**
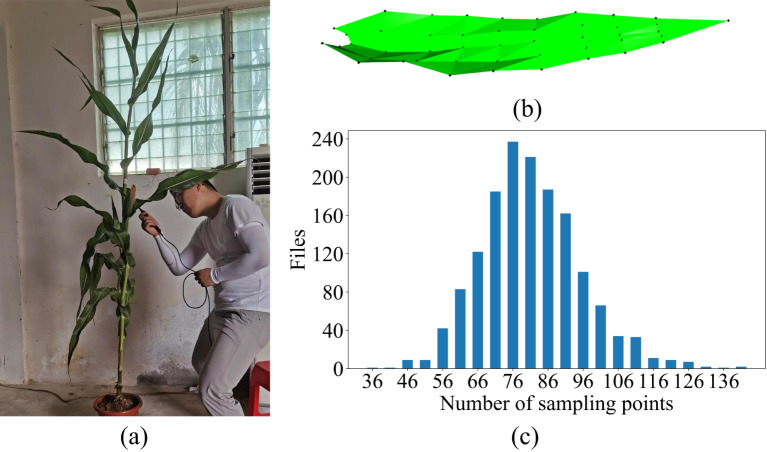
Raw data acquisition process and results. **(A)** Data acquisition, **(B)** the schematic of raw data acquisition, **(C)** statistics of the number of raw data points.

Data acquisition was carried out when the maize plants reached the silking stage, and the process was conducted from May 17 to May 27, 2021. The acquisition process was (1) Excavation of the root system and soil around the maize plants within a diameter of 25 cm and a depth of 20 cm, followed by arranging each individual plant in a pot (all sampling was done before 10 am) and transporting them indoors and watering them to minimize significant morphological changes caused by plant water deprivation (Wu et al., 2019). (2) 3D data of the ear leaves of each plant were collected by a digital probe using FastScan & FastRak 3D digitizers in combination with a Tx4 calibration transmitter. The 3D coordinates of selected leaf points were obtained manually (**Figure 1A**). The average acquisition time for each leaf was 5-8 minutes. If the plant had multiple ears, the leaf of the largest ear was selected as the ear leaf. Each data was checked visually to ascertain its accuracy. The data acquisition rule was to collect five points at even intervals using a digital probe starting from the leaf base perpendicular to the direction of the leaf midvein; then continue in the same manner upward along the direction of the leaf midvein to the tip of the leaf, with an unique point to indicate the tip of the leaf (**Figure 1B**). The number of points collected varied for different leaves due to different leaf lengths, but the number of points collected conformed to (5×n+1) as determined by the collection rules. The number of sampling points for most of the leaves ranged from 66 to 91 points, and the detailed sampling results are shown in **Figure 1C**.

A total of 518 distinct inbred lines were involved, with three samples collected for each inbred line. Finally, a total of 1,522 maize leaf model were obtained.

### 2.2 Overview

**Figure 2:**
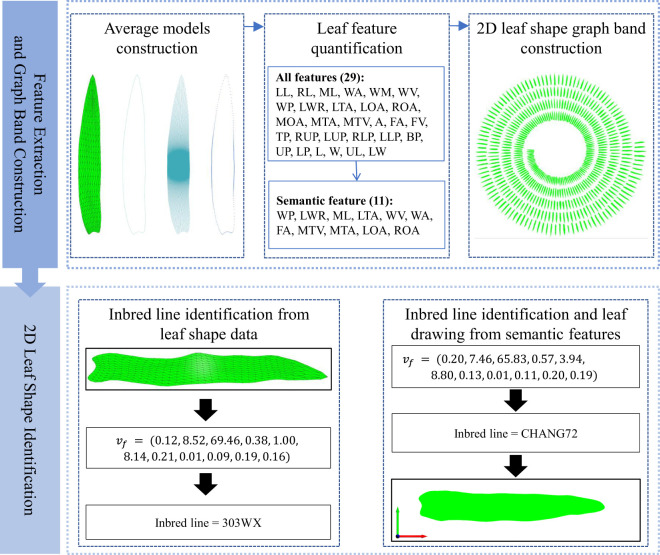
Overview of the overall process.

The general progression of the methodology is illustrated in **Figure 2**. The process begins with data acquisition and processing. Mesh subdivision and parameterization methods are employed to transform the original rough 3D mesh data acquired by the 3D digitizer into fine 2D mesh data. Subsequently, the average leaf shape models of each inbred line were constructed using the 2D leaf mesh model from the same inbred line. The morphological leaf features were quantified, then clustered and screened to determine the semantic features. A new phenotypic indicator *L*_2_ *_D_* that comprehensively reflect the 2D leaf shape was proposed and the 2D leaf shape atlas was constructed accordingly. For application, the atlas was used to identify the inbred line according to a given leaf, or drawing the 2D leaf shape according to a given semantic feature.

### 2.3 Data processing and morphological feature quantification of 2D leaf shapes in maize

#### 2.3.1 Maize leaf data preprocessing

The raw mesh data obtained by the 3D digitizer must undergo data preprocessing prior to its utilization in subsequent operations. The data preprocessing involves three steps: comparative examinations of the data within the same inbred line, data normalization, and raw mesh data subdivision.

##### 2.3.1.1 Comparative examinations of the data within the same inbred line

The objective is to ascertain the distinctions between the three samples for each of the 518 inbred lines, with the exclusion of samples exhibiting significant discrepancies. The four phenotypic features of leaf length, leaf width, aspect ratio, and raw mesh average angle were initially estimated based on the raw leaf mesh. The similarity was calculated based on the aforementioned four features, employing the Euclidean distance metric. The resulting similarity of the samples within the same inbred line were then compared. If there is sample with a similarity difference exceeding 0.1 with other samples within the same group, while the similarity difference between the remaining samples is less than 0.1, the sample exhibiting the greatest divergence is excluded. Duplicate groups with only two samples and one remaining sample were not subjected to screening. A total of 91 samples with excessive differences were excluded in this step, leaving 1,431 maize leaf data from 518 inbred lines for subsequent analysis.

##### 2.3.1.2 Data normalization

The principal axis direction of the maize leaf mesh model was initially obtained through principal component analysis (PCA). Subsequently, all principal axis directions were rotated so that the direction of the leaf tip pointed to the positive x-axis, with the center of mass designated as the origin [0, 0, 0]. Secondly, all leaves were maintained in a consistent proportion relative to one another, and the coordinate values of each point were uniformly scaled to the range of [-1, 1].

##### 2.3.1.3 Mesh subdivision

**Figure 3:**
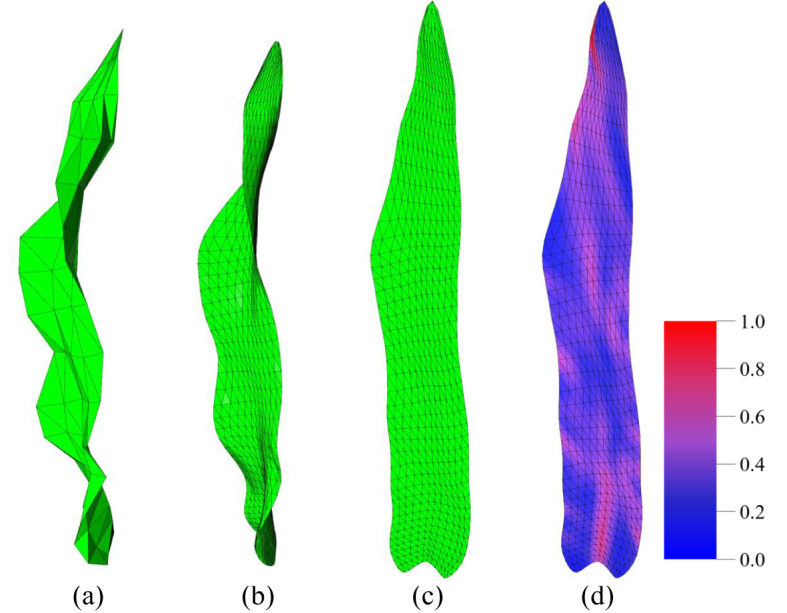
Illustration of key stages in data processing. **(A)** Raw 3D leaf data. **(B)** 3D leaf data after mesh subdivision (Kobbelt, 2000). **(C)** Parameterized 2D leaf mesh (Liu et al., 2008; Sorkine-Hornung and Alexa, 2007). **(D)** Fold visualization of a 2D leaf (more blue color means less folds, more red color means more folds).

The normalized mesh is then subdivided using the $\sqrt{3}$ (sqrt3) subdivision method (Kobbelt, 2000). The sqrt3 subdivision method is an efficient triangular mesh subdivision algorithm that belongs to the facet-splitting surface approximation mode. It has the advantages of a slower increase in mesh complexity during the refinement process and a certain degree of adaptive refinement. Following the subdivision of the raw leaf mesh, the resulting leaf model exhibits a shape that is more closely aligned with the actual leaf morphology (**Figure 3B**) compared to the raw data (**Figure 3A**). This enhanced resemblance to a more accurate reflection of the observed features. In this study, two iterations of the sqrt3 subdivision were performed on the original mesh. Following the operations, the number of triangular meshes was found to be nine times that of the original data, resulting in a more uniform and smooth mesh model.

#### 2.3.2 2D Flattening of 3D leaf mesh models

The process of flattening a 3D mesh model in 2D is referred to as planar parameterization. In this study, the as-rigid-as-possible (ARAP) (Liu et al., 2008; Sorkine-Hornung and Alexa, 2007) method is employed.

In the planar parameterization process, an energy function is used to delineate the discrepancy between the potential transformations and the target transformation.


$$E(u,\;L)=\sum_{t=1}^{T}A_{t}||J_{t}(u)-L_{t}|{|}_{F}^{2},$$

In the function, the area of the 3D triangles are $A_{t}\;(1\leq t\leq T)$ , For triangle *t*, $u_{t}=\{u_{t}^{0}\;,u_{t}^{1}\;,u_{t}^{2}\}$ denotes the 2D coordinates, and $x_{t}=\{x_{t}^{0}\;,x_{t}^{1}\;,x_{t}^{2}\}$ denotes the 3D coordinates. The relationship between $x_{t}$ and $u_{t}$ can be represented by a 2×2 Jobabian matrix $J_{t}(u)$ . $L_{t}$ is assigned as an auxiliary linear transformation ( 2×2 matrix). $||\cdot |{|}_{F}$ is the Frobenius norm. The variables in the energy function are the coordinates *u* of the mapping on the 2D plane and the transformation matrix $L_{t}$ . To minimize the energy function and restrict $L_{t}$ to the rotation matrix in the ARAP algorithm, the problem can be transformed into the following optimization problem after reconstruction and derivation:


$$(u,\;t)=argmin(u,\;t)E(u,\;t),\;L_{t}\in M$$

$$M=(\begin{array}{cc} \cos\theta & \sin\theta \\ -\sin\theta & \cos\theta \end{array}),\;\theta \in [0,2\pi )$$

This method maintains the angle of the triangles with the greatest possible constancy during the 3D to 2D mapping process, ensuring that the triangles are not distorted. Furthermore, the areas of the triangles are only slightly affected, thus preserving the invariance of the area to the greatest extent possible. Subsequently, the 3D mesh model is transformed into a corresponding 2D planar mesh model (**Figure 3C**). As illustrated in **Figure 3**, the planar parameterization of the 3D mesh model results in a deformed 2D mesh model due to the wrinkles and distortions inherent to the original 3D model. Consequently, the flattened 2D mesh model is not entirely symmetric along the axis of the leaf midvein. The 2D leaf model resulting from planar parameterization retains the basic phenotypic features of maize leaves, such as leaf length and width, to the greatest extent possible. Additionally, the deformations introduced by planar parameterization can also reflect certain 3D maize leaf phenotypic features, such as leaf twist. Accordingly, the 2D maize leaf mesh model obtained through the ARAP method is a more suitable means of extracting relevant phenotypic features for the analysis and research of leaf morphology.

#### 2.3.3 Construction of averaged 2D leaf models

Average leaf shape models were constructed for each of the distinct inbred lines of the 2D leaf mesh models. In total, 518 models were constructed. The leaf shape model is primarily concerned with the representation of leaf contour information, which is ultimately conveyed through a set of contour lines. The construction of the average model involves three steps: leaf contour extraction, contour sampling, and line set model construction.

##### 2.3.3.1 Leaf contour extraction

**Figure 4:**
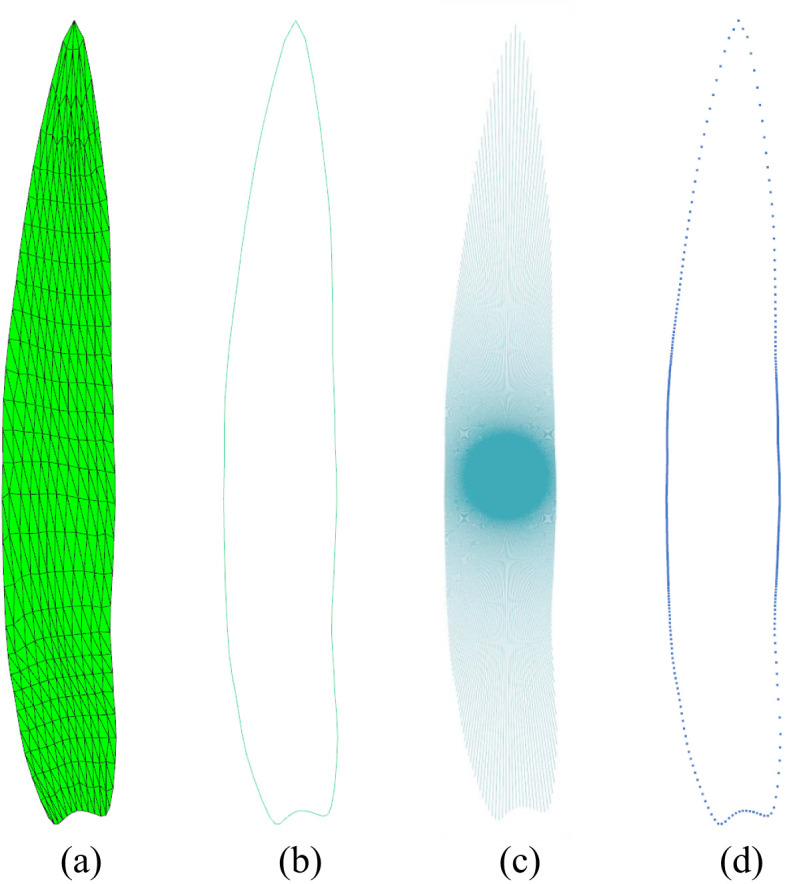
Leaf shape sampling. **(A)** Subdivided 2D maize leaf model. **(B)** Mesh contour. **(C)** Mesh contour sampling by emitting rays from the leaf center. **(D)** Sampled points of the leaf contour.

2D leaf mesh model is a non-closed mesh (**Figure 4A**), and the contour edges of the leaf mesh are the boundary edges. The edge contour is obtained by collecting all the boundary edges in the leaf mesh model. This process yields a line set comprising 1,431 leaves, as illustrated in **Figure 4B**.

##### 2.3.3.2 Contour sampling

A ray is initiated from the center point ([0, 0]) of the leaf and rotated around the center, intersecting with the leaf contour line set to obtain the sampling point set (**Figure 4C**). To guarantee the precision of the sampling, the sampling resolution was set to 600, thus ensuring the attainment of optimal sampling outcomes at the leaf tip and the base, which are situated at considerable distances from the center point (**Figure 4D**).

##### 2.3.3.3 Averaged 2D leaf model construction

The average point set of specific inbred line and the average point set of the entire leaves can be calculated from the sample results. The mean value of the coordinates of the sampling points at each position belonging to the same inbred line of samples is estimated. The overall leaf average point set is calculated in the same way. The points in the point set are connected sequentially in order to finally obtain the 518 inbred lines average model.

#### 2.3.4 Extraction of 2D leaf shape features from 2D mesh models

A total of 29 2D phenotypic leaf features were extracted from 1,431 2D mesh models of maize leaves, with reference to previous research (Wen et al., 2024a; Wu et al., 2022; Zheng et al., 2022). These features included 17 conventional phenotypic features and 12 leaf contour features. The conventional leaf parameters include: leaf length, leaf width, leaf tip angle, leaf area, etc. Additionally, the length of the left and right edges of the leaf, the offset degree of the left and right edges, and the width of the leaf at different positions were also extracted according to the 2D maize leaf model. The specific phenotypic features are presented in **Table 1**.

**Table 1:** Phenotypic features extracted from 2D maize leaf model.

ID	Feature name	Identifier	Explanation of the feature	Unit
1	Left length	LL	Length from the left point of the base part along the left margin to the point of the leaf tip	*cm*
2	Right length	RL	Length from the right point of the base part along the right margin to the point of the leaf tip	*cm*
3	Mid length (Leaf length)	ML	Length from the midpoint of the base part along the midvein to the point of the leaf tip (Leaf length)	*cm*
4	Width (average)	WA	The average width of the leaf obtained perpendicular to the direction of the midvein from the leaf base to the leaf tip	*cm*
5	Width (max)	WM	The max width of the leaf obtained perpendicular to the direction of the midvein from the leaf base to the leaf tip	*cm*
6	Width variance	WV	The variance of leaf width obtained perpendicular to the direction of the midvein from the leaf base to the leaf tip	*cm* ^2^
7	Widest position	WP	Distance from the position of maximum leaf width to the leaf base/leaf length (WP=0 if the position of maximum leaf width is at the leaf base; WP=1 if the position of maximum leaf width is at the leaf tip)	–
8	Length-width ratio	LWR	ML/WA	–
9	Leaf tip angle	LTA	The angle value between the leaf tip point and the neighboring points on both sides	*rad*
10	Left offset angle	LOA	Sum of the angle values between the lines between the points on the left edge of the leaf	*rad*
11	Right offset angle	ROA	Sum of the angle values between the lines between the points on the right edge of the leaf	*rad*
12	Mid offset angle	MOA	Sum of the angle values between the lines between the points on the midvein of the leaf	*rad*
13	Mid tortuosity (average)	MTA	Mean value of the angle between the line between the points on the midvein of the leaf and the line between the leaf base and the leaf tip.	*rad*
14	Mid tortuosity variance	MTV	The variance of the angle between the line between the points on the midvein of the leaf and the line between the leaf base and the leaf tip.	*rad* ^2^
15	Area	A	Total leaf mesh area	*cm* ^2^
16	Folding (average)	FA	Mean value of all triangular mesh angles of the leaf	*rad*
17	Folding variance	FV	The variance of all triangular mesh angles of the leaf	*rad* ^2^

**Figure 5:**
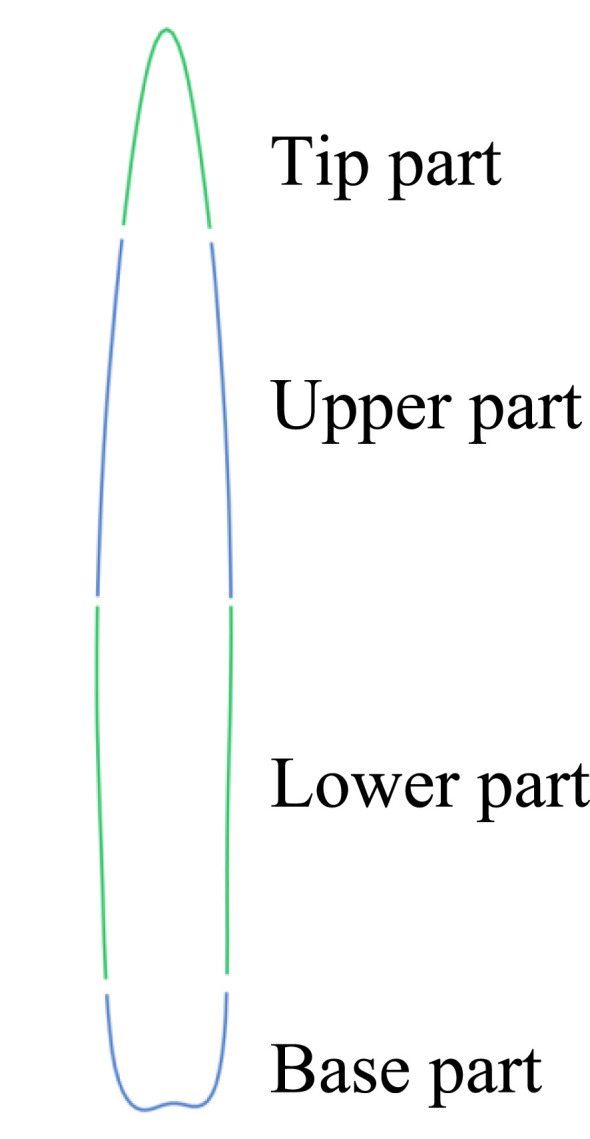
Leaf Contour Segmentation.

The remaining 12 features pertain to the leaf contour, as illustrated in **Table 2**. By dividing the leaf contour into six parts (**Figure 5**), namely, the tip part, the upper (left/right) part, the lower (left/right) part, and the base part, and calculating the distance between each part and the center point, a quantitative value of the leaf contour reflecting the shape of each part was obtained. The quantitative values of the various parts were used to obtain two additional features: the “Length/Width” and the “Upper - Lower”. This resulted in a total of 12 features.

**Table 2:** 2D leaf contour features.

ID	Feature name	Identifier	Explanation of the feature	
1	Tip part distance	TP	Mean distance of sampling points of the tip part from leaf center point	*cm*
2	Right upper part distance	RUP	Mean distance of sampling points of the upper right part from leaf center point	*cm*
3	Left upper part distance	LUP	Mean distance of sampling points of the upper left part from leaf center point	*cm*
5	Left lower part distance	LLP	Mean distance of sampling points of the lower left part from leaf center point	*cm*
6	Base part distance	BP	Mean distance of sampling points of the base part from leaf center point	*cm*
7	Upper part distance	UP	Mean distance of sampling points of the upper left part and upper right part from leaf center point	*cm*
8	Lower part distance	LP	Mean distance of sampling points of the lower left part and lower right part from leaf center point	*cm*
9	Length	L	Overall leaf length calculated from TP and BP correspondence	*cm*
10	Width	W	Overall leaf width calculated from RUP, LUP, RLP, LLP correspondingly	*cm*
11	Upper - Lower	UL	Difference between the widths of the upper and lower parts of the leaf calculated from the correspondence between UP and LP	*cm*
12	Length/Width	LW	Length width ratio of leaf calculated from L and W	–

##### 2.3.4.1 Calculation of 1D features (length, width and related features)

In the case of a 1D features, the calculation is based on the distance and angles between the points in the 2D mesh model. These 1D features include mid length (ML), maximum width (WM), average width (WA), variance, and widest position (WP), etc.

##### 2.3.4.2 Calculation of 2D features (area, leaf tip angle, offset and tortuosity)

Leaf area was calculated by summing the areas of all triangular mesh facets. Compared to traditional methods based on the formula (leaf length ×maximum leaf width × 0.75) (Valentinuz and Tollenaar, 2006) or (leaf length ×maximum leaf width × 0.765) (Lizaso et al., 2003), the approach of calculating the area of the mesh facets individually yields a more precise estimation of the leaf area.

Leaf tip angle, offset and tortuosity are angle features. The left and right edge angles at the leaf tip represents the leaf tip angle. The offset values of the left and right edges and midvein of the leaf are calculated by summing the angles obtained from the point-by-point line calculation of the mesh vertices at the corresponding positions. This emphasizes the overall degree of curvature of the leaf edge contour. The tortuosity of the midvein is calculated using the mean and deviation between the midvein and the line between the base to the tip of the leaf. It emphasizes the curvature of the leaf after the planar parametrization.

##### 2.3.4.3 Calculation of the folding degree

Folding is an important morphological feature of maize leaves, and a quantitative calculation method for folding based on data obtained by 3D digitizer was proposed. For the folding extracted from each sample, the average values of phenotypic features representing the value of each inbred line were obtained by intra-group averaging based on 518 different inbred lines. The FA mainly reflect the level of leaf folds comprehensively. The FA parameters were calculated as follows:


$$FA=\frac{\sum_{i=1}^{m}\frac{\sum_{j=1}^{n_{i}}\alpha ({\vec{N}}_{i},\;{\vec{N}}_{ij})}{n_{i}}}{m},\;$$

$$\alpha (\vec{x},\;\vec{y})=arccos(|\frac{\vec{x}\cdot \vec{y}}{\left|\vec{x}|\cdot |\vec{y}\right|}|)$$

where *m* represents the total number of mesh patches in the maize leaf mesh model, $n_{i}$ represents the number of patches adjacent to patch *i*, ${\vec{N}}_{i}$ represents the normal vector of the patch *i*, ${\vec{N}}_{ij}$ represents the normal vector of the $j_{\text{th}}$ patch neighboring patch *i*.

### 2.4 Maize leaf semantic feature extraction and 2D leaf shape atlas construction

#### 2.4.1 2D leaf shape semantic feature determination

While the aforementioned 29 features provide comprehensive coverage of leaf shape, some features exhibit similarities to a certain extent. This correlation between features may result in redundancy, which could impede the simplicity of the feature representation. To address this, we have selected a subset of features as the semantic features of the 2D leaf shape, aiming to maximize feature coverage while simplifying the representation.

The correlation and clustering analyses were conducted using the 29 2D leaf shape features obtained above to determine the key features. The hierarchical clustering approach employs a specified method to quantify the degree of affinity between the features. The process involves initially clustering the more closely related features into one class, and then repeating this until all the features are clustered into one class. The selection of a suitable distance metric is paramount for accurately measuring the difference between phenotypic features. In this study, Pearson Correlation Coefficient is employed as the distance metric, which reflects the degree of linear correlation between the two features under investigation. The Pearson Correlation Coefficient is calculated as follows:


$$1-\frac{\left(u-\bar{u})\cdot (v-\bar{v}\right)}{||u-\bar{u}|{|}_{2}||v-\bar{v}|{|}_{2}}$$

where $\bar{v}$ is the mean of the elements of vector v, and x·y is the dot product of *x* and *y*. A high correlation between two phenotypic features indicates a significant degree of overlap and similarity in their ability to reflect leaf phenotype. Consequently, the more representative features were selected for characterization, with the objective of simplifying and refining the feature information. Furthermore, Average Linkage is chosen as the linkage algorithm, as it has been demonstrated to effectively calculate the average distance between all pairs of points in two clusters, thereby facilitating the generation of compact clusters. The Average Linkage (average) algorithm assigns:


$$d(u,\;v)=\sum_{ij}\frac{d(u[i],\;v[j])}{\left(|u|*|v|\right)}$$

for all points *i* and *j* where |u| and |v| are the cardinalities of clusters *u* and *v*, respectively.

#### 2.4.2 2D leaf shape atlas construction

Once the semantic features of the 2D leaf shape have been determined, a n-dimensional feature vector $v_{f}$ can be constructed by weighting and combining all the semantic features to reflect the main characteristics of each leaf. The feature vectors were used to sequencing the 518 inbred lines of leaves according to the differences in the features. In this sequencing process, phenotypic features should be comprehensively considered. The sorted result was used to construct a atlas for 2D leaf shape, which reflected the differences in the leaf shapes of the maize in comparison with the evolutionary process.


$$v_{f}\;=\;(f_{1},f_{2},\cdots ,\;f_{n})$$

$$w\;=\;(w_{1},\;w_{2},\cdots ,\;w_{n})$$

where $f_{i}$ is the $i_{th}$ semantic feature and $w_{i}$ is the weight coefficient of the $i_{th}$ feature.

In the determination of w , it should be noted that the number of inbred lines included in this research is 518, with the data for each inbred line consisting of only one to three samples. Given the limited number of samples within each group, it is challenging to achieve a superior training outcome when the sample size is insufficient utilizing machine learning techniques. Consequently, when determining the optimal weights, we employ the greedy algorithm to enumerate and calculate the most suitable weights for each semantic feature, and then as the weighting coefficients for the final leaf feature vector.

### 2.5 Inbred line identification based on 2D leaf shape atlas

Accurately identify a given inbred line based on the observed leaf shape data is a crucial aspect of crop breeding, representing one of the practical applications of the proposed method. The process of identifying a inbred line based on 2D leaf shape atlas can be divided into two main aspects: identifying the inbred line from the given leaf, and identifying the 2D leaf shape from the given semantic features.

#### 2.5.1 Inbred line identification from given leaf shape data

The cosine similarity is a difference measure between two vectors in an n-dimensional space. A feature vector was constructed for each 2D mesh model of a maize leaf based on the semantic features of the leaf. Given an example leaf shape data, the cosine similarity was calculated by comparing the vector of this example similar leaves in the atlas. The inbred line with the highest similarity was selected and identified as the inbred line of the given leaf.

#### 2.5.2 Inbred line identification and leaf shape drawing from given semantic feature

As with the aforementioned method for identifying a given leaf shape, the closest maize inbred line to a given feature vector is determined by calculating the cosine similarity. This allows the leaf shape of that inbred line to be identified in the constructed leaf shape atlas, which in turn permits the unique drawing (visualization) of the 2D leaf shape for a given semantic feature.

#### 2.5.3 Evaluation metric

The Top-X Accuracy metric $r_{Top-X}$ , is employed to evaluate the performance of inbred line identification from either a specific 2D leaf shape or a semantic feature vector. The value of $r_{Top-X}$ indicates that the ground truth inbred line labels of the leaf instance data are within the top *X* of the predicted similarity results. In this context, $r_{Top-1}$ indicates that the inbred line with the highest predicted similarity is the ground truth. $r_{Top-3}$ indicates that the ground truth is among the top three results for similarity.

## 3 Results

### 3.1 Results and analysis of 2D flattening from 3D leaves

**Figure 6:**
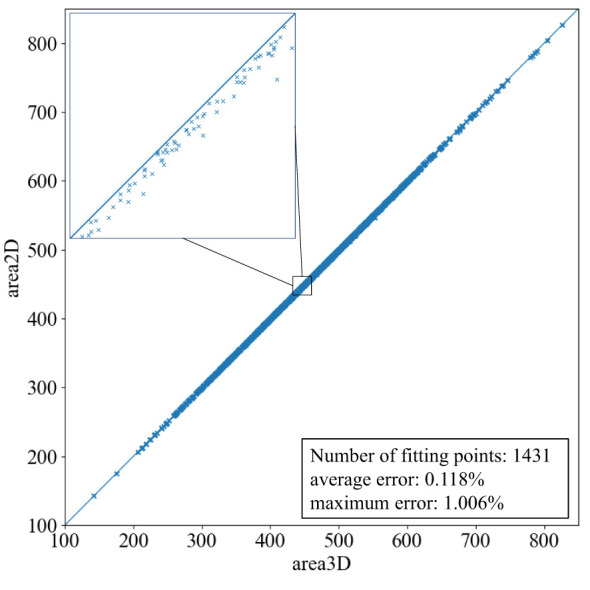
Leaf area ratio of 2D model to 3D model after planar parameterization.

The ARAP planar parameterization method is designed to minimize changes in mesh area and shape distortion during 2D flattening. A comparison of the area of 3D and 2D mesh models before and after planar parameterization indicates that the area of the mesh models after planar parameterization is generally reduced by a very small amount. The average leaf area ratio of the 2D mesh models to the 3D mesh models for the 1,431 leaf mesh models is 99.88%, and there is only a change of 0.12%. As illustrated in **Figure 6**, the area ratio of the 2D and 3D mesh models is consistently above 0.990 and below 1.000. The majority of the data points are situated within the interval between 0.998 and 1.000, with only a few instances where the ratio is below 0.998.

**Figure 7:**
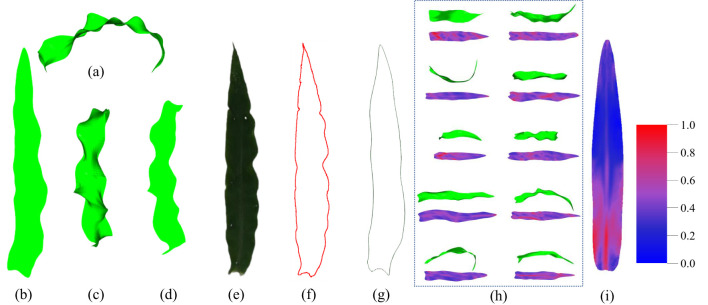
Comparison of the results of the planar parameterization. **(A)** 3D mesh model of the maize leaf. **(B)** 2D mesh model after ARAP planar parameterization. **(C)** Front view of the 3D mesh model of the maize leaf. **(D)** Front view projection of the 3D mesh model. **(E)** Top view image of a manually flattened maize leaf. **(F)** Extracted leaf contour of **(E)**. **(G)** Extracted leaf contour after ARAP flattened. **(H)** The result of planar parameterization for different leaves. **(I)** Fold visualization of the average model for all leaves (more blue means less folds, more red means more folds).

As illustrated in **Figure 7**, the ARAP planar parameterization technique is capable of accurately retaining the morphology of the 3D leaf mesh, as evidenced by the zigzagging of the 2D leaf mesh edges, while the projection method is susceptible to influences such as the projection angle. The ARAP planar parameterization technique also accurately restores the morphometrics of the entire leaf, including the tip and the base. In contrast, the 2D mesh based on the projection method is unable to accurately reproduce the leaf tip and the base due to the excessive curvature of the leaf. Additionally, the side edges of the 2D mesh lack smoothness due to the mutation of edge contour caused by the projection, which hinders the accurate reflection of the true shape of the original 3D leaf. It is challenging to accurately represent the true shape of the original leaf. The extracted leaf contour, obtained by manually flattening the leaf and photographing it, is shown in **Figure 7F**. Due to the inability to eliminate wavy folds of the leaves when flattening the leaves manually, excessive wavy zigzags can be observed in the extracted contour. In comparison, the contour obtained by the ARAP method exhibits a smoother contour and circumvents substantial errors in estimating phenotypic features such as leaf area.

Furthermore, the 2D mesh model, following ARAP planar parameterization, can effectively visualize and demonstrate the folds of the 3D mesh model (**Figures 3B**, **7H**). The visualization of 3D leaf folds on a 2D leaf after planar parameterization reveals that the red regions correspond to larger folds. Firstly, it can be observed in **Figure 7H** that the majority of leaves exhibit a large percent red area in the base midline portion, this distinct feature is also evident in **Figure 7I**. This phenomenon can be attributed to the bending morphology exhibited by ear leaves, which is evident from the base of the leaf midvein to the leaf sheath connection. Given that ear leaves are longer and wider, this bending structure is more pronounced, providing greater support for the leaves. It should be noted that, with the exception of the base of the leaf, the folds can be observed in the position of the midvein. This is due to the fact that all the leaves exhibit a bent morphology at the position of the leaf midveins. Secondly, as illustrated in **Figure 7I**, the folds of the maize leaves are predominantly concentrated in the lower middle portion of the leaf, where the overall distribution of the folds is characterized by a dense area with a curved boundary. This boundary is a consequence of the widespread bending of the left and right edges of the middle and lower parts of the leaf towards the back of the leaves, as well as the pronounced wavy folds of the leaf perpendicular to the direction of the leaf veins in this region. Thirdly, a specific distribution of folds is observed at the tip of the leaf. This distribution is not so dense than that observed at the lower middle portion of the leaf, primarily due to the overall amplitude of the folds at the tip being smaller than that observed at the lower middle portion of the leaf. The formation of folds at the tip of the leaf can be attributed to the fact that the tip is more susceptible to rotation and deflection than other regions. At last, the upper-middle region exhibits the lowest degree of folding when compared to other regions. Except the bend of the midveins in the middle region displays a notable degree of folding, the surface of this portion is more smoothed than that of the remainder.

### 3.2 Semantic feature of 2D leaf shape

**Figure 8:**
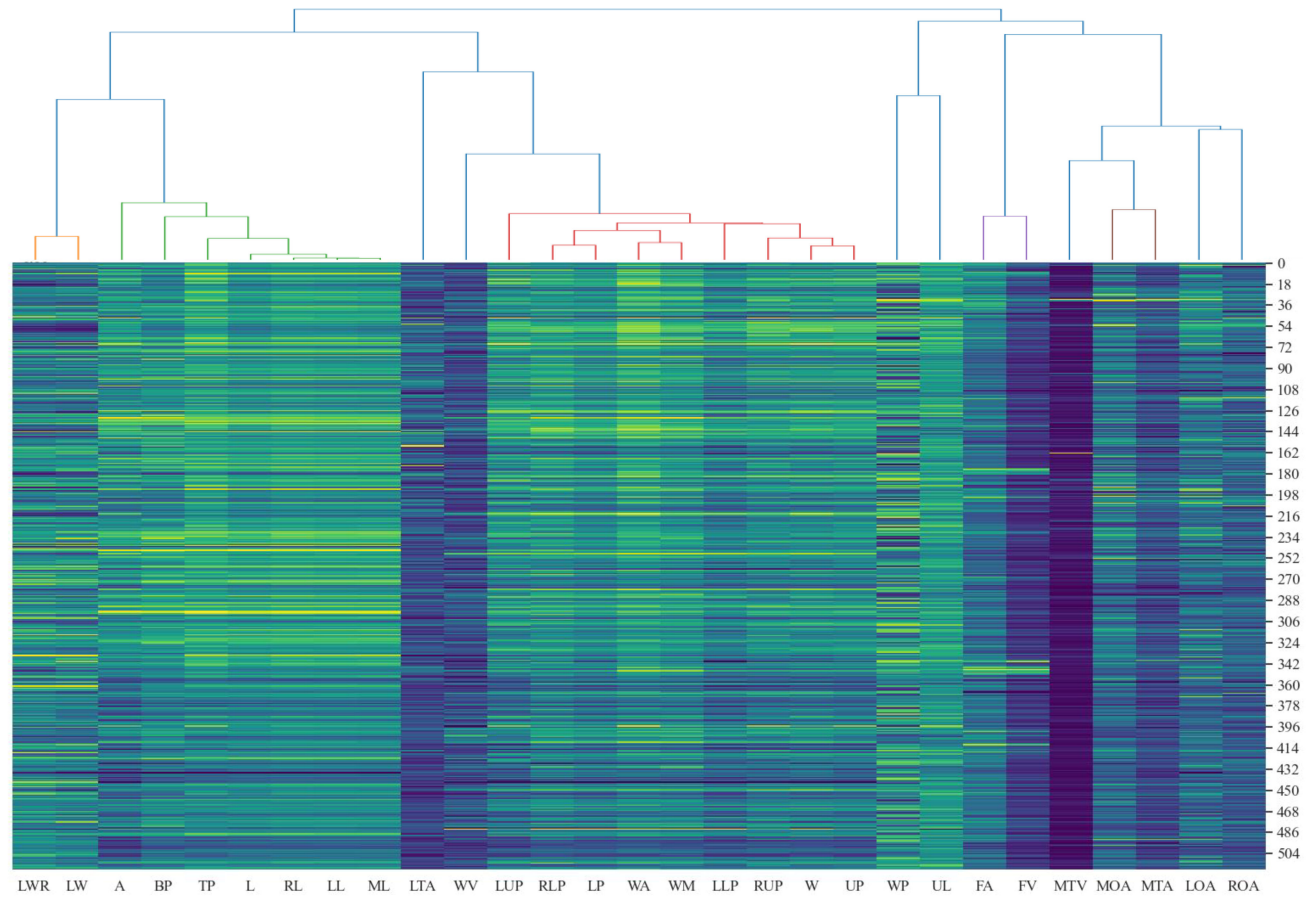
Cluster analysis of 2D leaf shape features.

11 out of 29 2D leaf-shape features are identified as semantic features (WP, LWR, L, LTA, WV, W, FA, MTV, MTA, LOA, ROA) by setting the clustering threshold to 0.25 and using the “average” method and the “correlation” metric in hierarchical clustering, as shown in **Figure 8**. The determination of an optimal clustering threshold is a critical step. Through experimental analysis, a threshold value of 0.25 has been determined to achieve optimal distinction between phenotypic features based on their correlation. That is, most of the 2D leaf-shape characteristics can be characterized using these 11 semantic features.

**Figure 9:**
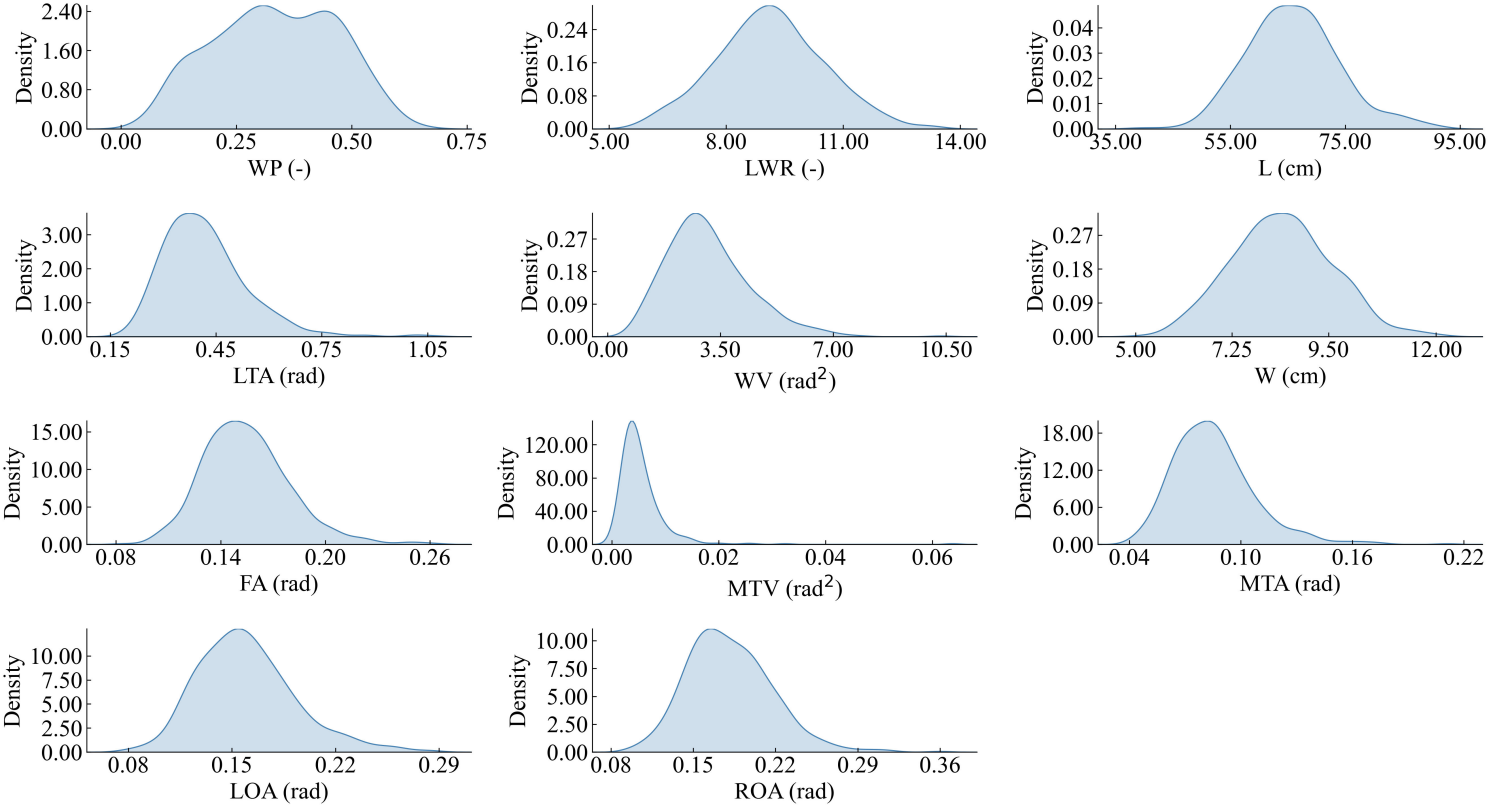
Distribution function of the 11 semantic features.

**Figure 9** depicts the fitted distribution functions of the 11 semantic features. To determine the weights of each semantic feature, seven values were taken from the interval of [0, 3] with step length of 0.5 for each semantic feature to validate the optimal weight. We sequentially traversed to test the identification ability corresponding to seven different weights with the weights of the other semantic features had been set to 1, then we selected the weight with the best identification ability to be determined as the corresponding weight of the semantic feature. The 11 semantic features were systematically traversed to ascertain the optimal weights, and a set of optimal weights is determined. Following the validation and normalization of the features, the correspondence between the semantic features and the corresponding optimal weights is presented in **Table 3**. Notably, L, which reflects the length of the leaf, plays a pivotal role in the identification process. Additionally, the two features, MTA and MTV, measure the degree of tortuosity of the leaf and the variation degree of tortuosity, both of which are crucial for identify between different leaf shapes. Additionally, the W reflects the width of the leaf. Consequently, the aforementioned four parameters are assigned the highest weights.

**Table 3:** Semantic features with corresponding weights.

ID	Identifier	Physiological significance	Weight
1	WP	Widest position of leaf	0.028
2	LWR	Leaf aspect ratio	0.056
3	L	Average leaf length based on leaf contour	0.167
4	LTA	Leaf tip angle	0.111
5	WV	Variance of the width of the leaf	0.028
6	W	Average leaf width based on leaf contour	0.111
7	FA	Fold	0.083
8	MTV	Variance of leaf midvein tortuosity	0.139
9	MTA	Mean value of leaf midvein tortuosity	0.167
10	LOA	Degree of curvature of the left edge of the leaf	0.056
11	ROA	Degree of curvature of the right edge of the leaf	0.056

### 3.3 Results of 2D leaf shape atlas construction

In accordance with the methodology in section 2.4.2, the semantic feature vectors of 2D leaf shape can be determined by the 11 semantic features with the corresponding weights. The complete semantic feature vectors $v_{f}$ and the corresponding weight vectors w are as follows:


$$\begin{array}{c} v_{f}\;=\;(WP,\;LWR,\;L,\;LTA,\;\\ WV,\;W,\;FA,\;MTV,\;MTA,\;LOA,\;ROA) \end{array}$$

$$\begin{array}{c} w\;=\;(0.028,\;0.056,\;0.167,\;0.111,\;0.028,\;0.111,\;\\ 0.083,\;0.139,\;0.167,\;0.056,\;0.056) \end{array}$$

To establish a rule for ranking the 2D leaf shapes, a weighted vector $v_{f\_w}$ is defined using the feature vector $v_{f}$ with the weight vector w . $L_{2D}=||v_{f\_w}||$ , the modulus of the weighted vector $v_{f\_w}$ , is then calculated as the rule for ranking the 2D leaf shape of all the inbred lines, thus generating a atlas. $L_{2D}$ serves as a comprehensive indicator reflecting the overall 2D morphology features of a maize leaf. $L_{2D}$ increases along with the semantic characteristics of the leaf become more pronounced (e.g., longer length, wider width, more curved edges, etc.). Conversely, $L_{2D}$ also decreases as the phenotypic characteristics of the leaf become less pronounced.


$$L_{2D}=||v_{f\_w}||=\sqrt{\sum_{i=1}^{n}(w_{i}\cdot v_{f\_i})}$$

$$\sum_{i=1}^{n}w_{i}=1$$

where $w_{i}$ is the $i_{th}$ parameter in the weight vector w , and $v_{f\_i}$ is the $i_{th}$ parameter in the feature vector $v_{f}$ .

**Figure 10:**
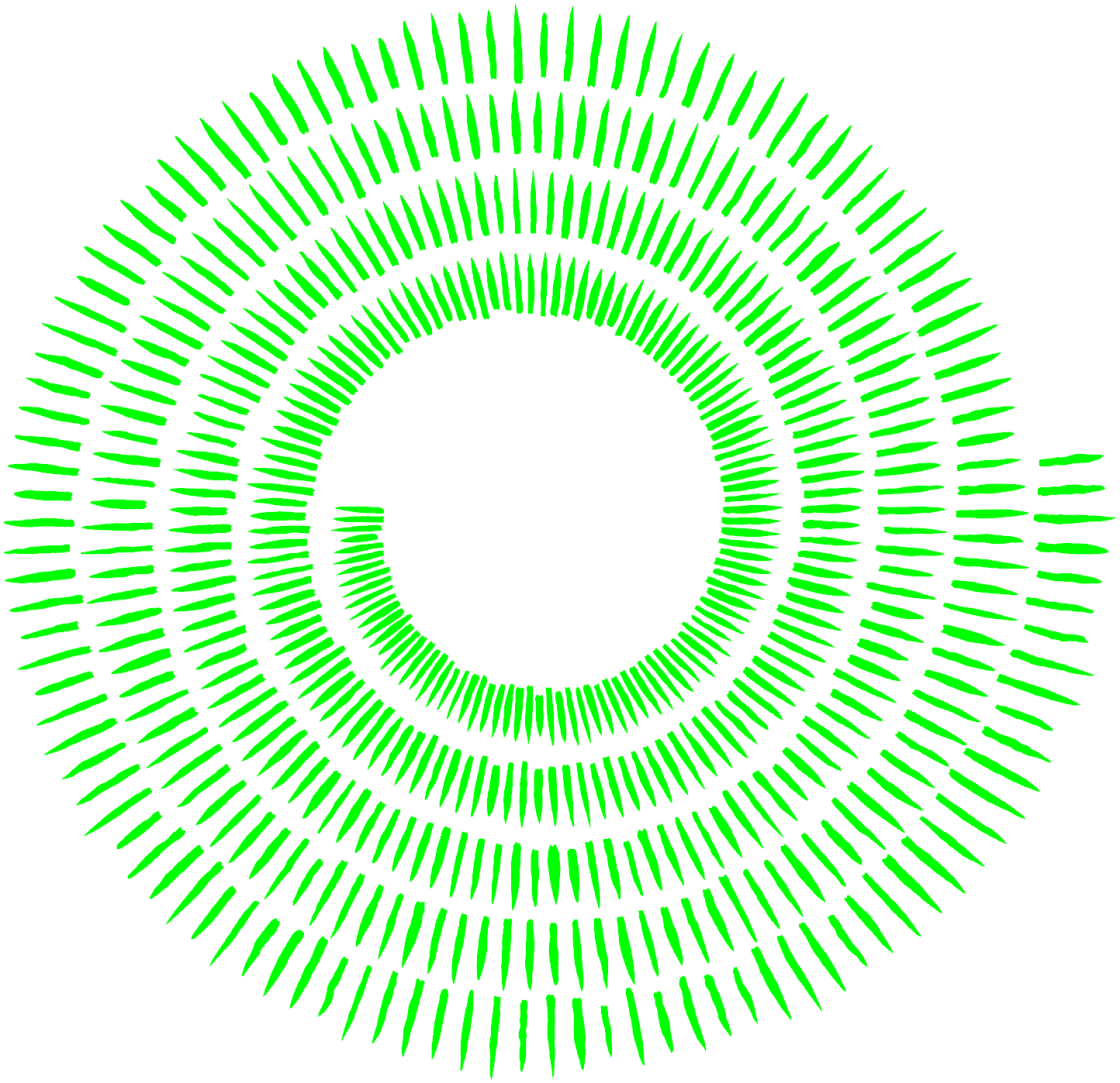
2D leaf shape atlas ranked using $L_{2D}$ ( $L_{2D}$ increasing from inside to outside).

The 518 inbred lines were ranked according to the $L_{2D}$ to form the final 2D leaf shape atlas (**Figure 10**). As illustrated in **Figure 10**, the maize leaves in the inner circle are relatively diminutive in both overall length and width, and the leaf contour is relatively smooth, exhibiting no discernible zigzagging at the edges. In contrast, the leaves in the outer circle are larger in both overall length and width, displaying pronounced zigzagging at the edges, and the phenotypic characteristics of the leaves are more pronounced.

### 3.4 2D leaf shape identification results

#### 3.4.1 Inbred line identification results for given leaf shape

**Figure 11:**
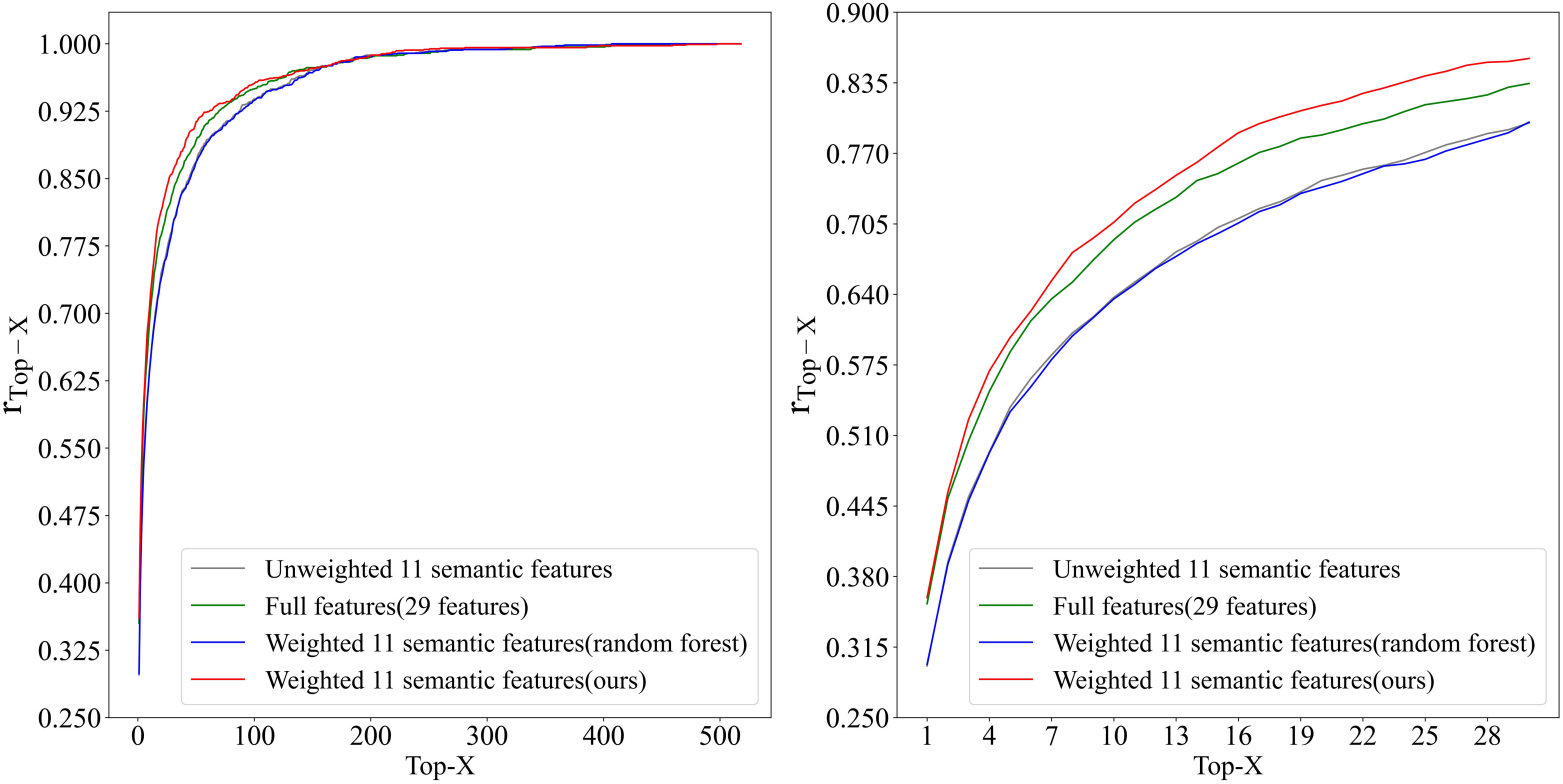
Top-X Accuracy trend in inbred line identification using leaf shape features.

A total of 1,431 leaves were utilized to conduct inbred line identification, and using $r_{Top-X}$ as the performance evaluator. For comparison, experiments were conducted in three setups: (1) using the full 29 features, (2) using unweighted 11 semantic features, and (3) using 11 semantic features with trained weights according to the random forest. The results of the experiment are presented in **Figure 11** and **Table 4**. The weighted features, calculated using the greedy algorithm, demonstrated the optimal performance. Furthermore, the proposed method outperforms the other three feature vector design methods in $r_{Top-1}$ , and the accuracy remains superior as X increases. The $r_{Top-X}$ of this method exhibited accelerated improvement with increasing of X, attaining $r_{Top-10}$ of 0.706, $r_{Top-20}$ of 0.810, and $r_{Top-45}$ of 0.900. Due to the considerable number of inbred lines included in this study (a total of 518), $r_{Top-10}$ has been possible to identify the inbred lines within 2% of the total number of inbred lines. Furthermore, $r_{Top-20}$ and $r_{Top-45}$ have been achieved, whereby the inbred lines have been identified within 4% and 9% of the total number of inbred lines, respectively. Given the limited number of samples obtained from the various inbred lines and the observed variability in the characteristics of the leaves, the outcomes of this study are noteworthy.

**Table 4:** The $r_{Top-X}$ comparison obtained using the four methods.

$r_{Top-X}$	Unweighted 11 semantic features	Full features (29)	Weighted 11 semantic features (random forest)	Weighted 11 semantic features (ours)
$r_{Top-1}$	0.298	0.355	0.299	**0.361**
$r_{Top-2}$	0.393	0.452	0.391	**0.458**
$r_{Top-3}$	0.454	0.505	0.450	**0.525**
$r_{Top-4}$	0.495	0.551	0.494	**0.570**
$r_{Top-5}$	0.536	0.587	0.532	**0.600**
$r_{Top-6}$	0.563	0.616	0.555	**0.625**
$r_{Top-7}$	0.584	0.636	0.580	**0.653**
$r_{Top-8}$	0.604	0.651	0.602	**0.679**
$r_{Top-9}$	0.619	0.672	0.618	**0.692**
$r_{Top-10}$	0.637	0.690	0.636	**0.706**

Optimal performance is indicated by the use of bold text.

#### 3.4.2 Identification results for given semantic features

**Figure 12:**
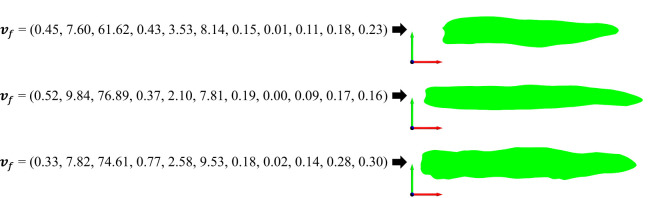
Three examples of generating and drawing 2D leaf models from feature vectors.

For semantic features of a 2D leaf, the model with the highest weighted cosine similarity among the 518 inbred line models can be identified as the one that most closely aligns with and represents the corresponding 2D model of the leaf (**Figure 12**). To guarantee that the leaf illustration accurately reflects the semantic features, the matching threshold is set to 0.9. This implies that when the highest match between a specific semantic feature and the inbred line model library is less than 0.9, it is assumed that this particular structure of a maize leaf does not exist, and thus, the specified maize leaf model cannot be generated. This is a straightforward method for generating 2D leaf models based on feature matching. The data template library in this study provides comprehensive coverage of maize 2D leaf models, and the feature vectors constructed in this study encompass the majority of features observed in maize 2D leaf models. Furthermore, the 518 inbred line models are all generated based on true leaves, ensuring that the generated results are rich, authentic, and of significant research value.

## 4 Discussion

### 4.1 Leaf shape atlas promotes plant phenotypic identification

This study benefits from “human face recognition” technology to identify and categorize 2D maize leaves. The key point is that to quantify the morphological features of 2D leaf, and ascertain the typical characteristics as semantic features, which then allows for focused exploration and classification. In comparison to existing research on crop leaf shapes and phenotypic identification, this study presents the following novel contributions and advances:

(1) Construction method of leaf 2D mesh model. This study applied the ARAP planar parameterization in converting 3D mesh model to 2D planar mesh. The method allows for the more precise retention of the 3D structure, surpassing the traditional methods such as projection or manual flattening (Wang et al., 2022). Furthermore, the method avoids the potential loss of information due to projection angle (**Figures 7B, D**). For example, the leaf area is highly accurately reserved in the 2D mesh model obtained by ARAP planar parameterization method.
(2) Quantification and determination of semantic leaf features. A multitude of phenotypes for expressing leaves have been put forth in previous studies. However, the divergence of research directions have resulted in numerous redundancies of phenotypic features. For example, the terms “upper leaf midrib sag” and “leaf midrib curvature” in the work of Wu et al. (2022) were used to describe the curvature of the leaf midrib from different perspectives. Zheng et al. (2022) put forth the terms “leaf sag” and “leaf bending” to describe the sagging curvature and overall bending curvature of the leaf, respectively. The two aforementioned metrics exhibit a high degree of similarity. Consequently, the ability to effectively screen the features, ensuring both concise representation and comprehensive coverage, becomes a pivotal determinant of the success of subsequent research. In this study, a hierarchical clustering method was employed to screen and categorize the phenotypic features proposed in previous studies, resulting in the determination of 11 parameters of 2D maize leaf features that were not duplicated by each other and could be more comprehensively covered. This approach enabled the condensation and compression of the 2D leaf features, laying the foundation for the construction of feature vectors and the identification of leaf cultivars or inbred lines.
(3) 2D leaf shape atlas construction. Given the considerable number of phenotypic features employed to describe leaf shapes, a comprehensive ranking of all leaf shapes is essential to fully reflect the observed variation between leaf shapes. In previous studies (Åström et al., 2015; Cohen, 2011; Dubey et al., 2024), the screening of features are remain qualitative, and comprehensive quantitative ranking integration is lacking. In this study, a comprehensive 2D leaf shape indicator *L*_2_ *_D_* was obtained by weighting the 11 semantic features and estimating the module length of the vector. The 2D leaf shape atlas was obtained by ranking the 2D leaves according to *L*_2_ *_D_*. This approach allows for sequencing leaf shapes based on the main leaf features. Additionally, it demonstrates the relationship between the expression of the 2D leaf shape features, from weak to strong, in a population comprising a vast array of inbred lines.
(4) Inbred line identification and 2D leaf shape generation. In contrast to previous identification studies, which have been conducted across species (Kumar et al., 2019) and plants with substantial variation (Dubey and Thanikkal, 2023), the identification of the inbred lines to which a given leaf shape belongs has enabled phenotyping to reach “cultivar-level resolution,” which is crucial for the advancement of phenotyping. The creation of leaf shape models based on semantic features is a highly interpretable process, and this study is of great significance in promoting the generation of both 2D and 3D models based on semantic features. This study presents a research pipeline for the analysis of crop phenotypic big data and the generation of models based on phenotypic big data. The pipeline includes feature extraction, semantic feature extraction and screening, construction of comprehensive indexes and atlas, and inbred line identification based on atlas. It offers novel methodologies and strategies for the extraction of knowledge from phenotypic big data, as well as a comprehensive workflow that serves as a valuable reference for the identification of plant cultivars.

### 4.2 Limitations and future works

The study focuses on the process of analyzing 2D shape of maize leaves, with less attention on the 3D morphology and related analyses. In subsequent studies, the identification based on 2D features will be expanded to 3D features. The feature extraction and quantitative analysis will be conducted based on the 3D mesh of maize leaves. The phenotypic features obtained from the 2D and 3D meshes will be combined to realize more efficient and accurate variety identification. Additionally, further studies on the physiological properties of maize leaves, such as light interception capacity (Song et al., 2023) and the relationship between phenotype, genes, and environment, based on the 3D structure, will be conducted. For instance, the correlation between leaf phenotype and genome through GWAS will be conducted. The study also has the advantage of non-destructive acquisition of 3D models of leaves and extraction of phenotypic features. The substantial number of samples collected ensures comprehensive coverage of leaf shape diversity, and the results indicate that the 3D flattening method is area-preserving. Consequently, the method should be applied in leaf monitoring at different growth stages.

Moreover, the 2D leaf shape generation method employed in this study, while maintaining interpretability, requires further enhancement through the integration of deep learning and other techniques to augment the degree of freedom and flexibility in generation. This study serves as an initial investigation of deep learning-based 2D leaf generation, with the objective of providing accurate leaf parameter metrics and 2D leaf shape reference standards for subsequent studies. Subsequently, we will employ a deep learning-based approach to achieve the objective of 2D leaf shape generation by integrating techniques associated with generative AI, such as the diffusion model and CLIP. Concurrently, upon completion of the analysis and research of the 3D maize leaf shapes, in conjunction with the findings of the 2D models generation, the generation of 3D maize leaves with a sense of realism will be pursued as the ultimate objective of the research.

## Data Availability

The data analyzed in this study is subject to the following licenses/restrictions: The data will be made available upon request. Requests to access these datasets should be directed to guoxy73@163.com.
